# Genomic insight into the scale specialization of the biological control agent *Novius pumilus* (Weise, 1892)

**DOI:** 10.1186/s12864-022-08299-w

**Published:** 2022-01-31

**Authors:** Xue-Fei Tang, Yu-Hao Huang, Hao-Sen Li, Pei-Tao Chen, Huan-Ying Yang, Yuan-Sen Liang, Xue-Yong Du, Zhen-Hua Liu, En-Feng Li, Yu-Chen Yang, Hong Pang

**Affiliations:** 1 State Key Laboratory of Biocontrol, School of Life Sciences / School of Ecology, Sun Yat-sen University, Guangzhou, Guangdong China; 2 School of Biological Sciences, University of Queensland, Brisbane, Queensland Australia

**Keywords:** Genome, Transcriptome, Biological control, Ladybird, *Novius*, *Novius pumilus*, Prey adaptation

## Abstract

**Background:**

Members of the genus *Novius* Mulsant, 1846 (= *Rodolia* Mulsant, 1850) (Coleoptera, Coccinellidae), play important roles in the biological control of cotton cushion scale pests, especially those belonging to *Icerya*. Since the best-known species, the vedalia beetle *Novius cardinalis* (Mulsant, 1850) was introduced into California from Australia, more than a century of successful use in classical biological control, some species of *Novius* have begun to exhibit some field adaptations to novel but related prey species. Despite their economic importance, relatively little is known about the underlying genetic adaptations associated with their feeding habits. Knowledge of the genome sequence of *Novius* is a major step towards further understanding its biology and potential applications in pest control.

**Results:**

We report the first high-quality genome sequence for *Novius pumilus* (Weise, 1892), a representative specialist of *Novius*. Computational Analysis of gene Family Evolution (CAFE) analysis showed that several orthogroups encoding chemosensors, digestive, and immunity-related enzymes were significantly expanded (*P* < 0.05) in *N. pumilus* compared to the published genomes of other four ladybirds. Furthermore, some of these orthogroups were under significant positive selection pressure (*P* < 0.05). Notably, transcriptome profiling demonstrated that many genes among the significantly expanded and positively selected orthogroups, as well as genes related to detoxification were differentially expressed, when *N. pumilus* feeding on the nature prey *Icerya* compared with the no feeding set. We speculate that these genes are vital in the *Icerya* adaptation of *Novius* species.

**Conclusions:**

We report the first *Novius* genome thus far. In addition, we provide comprehensive transcriptomic resources for *N. pumilus*. The results from this study may be helpful for understanding the association of the evolution of genes related to chemosensing, digestion, detoxification and immunity with the prey adaptation of insect predators. This will provide a reference for future research and utilization of *Novius* in biological control programs. Moreover, understanding the possible molecular mechanisms of prey adaptation also inform mass rearing of *N. pumilus* and other *Novius*, which may benefit pest control.

**Supplementary Information:**

The online version contains supplementary material available at 10.1186/s12864-022-08299-w.

## Background

Ladybirds (Coleoptera, Coccinellidae) are a group with diverse feeding habits. Most of them are specialist feeders, while some species have a much wider range of prey [1]. Evolutionary studies have suggested that the ancestors of modern ladybirds switched from mycophagy to scale insects feeding, and some species have even shifted the feeding behaviors to plants, mites, whiteflies and aphids [2, 3]. However, *Novius* Mulsant, 1846 (= *Rodolia* Mulsant, 1850) (Coleoptera, Coccinellidae) still maintains the characteristics of specifically feeding on scale pests. Beetles in the *Novius* are probably best known for their extensive use as biological control agents, mainly targeting cotton cushion scale species (Margarodidae), especially those belonging to *Icerya* (hereafter we use *Icerya* and cottony cushion scale interchangeably) [4, 5]. One example is that the introduction of the vedalia beetle, *Novius cardinalis* (Mulsant, 1850), from Australia to California achieved great success in controlling the cottony cushion scales, *Icerya purchasi* Maskell, 1879, which was a milestone in contemporary biological control [6, 7].

Another famous member in *Novius*, *Novius pumilus* (Weise, 1892) (=*Rodolia pumila* Weise, 1892), has also been widely used in biocontrol for *I. aegyptiaca* and *I. seychellarum* in Spain, Peru and the islands of Micronesia, etc. [8, 9]. *N. pumilus* is native to the East, and very common in southern China [10]. Similar to *N. cardinalis*, both adults and larvae of *N. pumilus* mainly prey on *Icerya* pests, including *I. purchasi* Maskell, *I. seychellarum* (Westwood), and *I. aegyptiaca* Douglas. Female adults of *N. pumilus* usually lay their eggs in exposed sites in the vicinity of prey; otherwise, they oviposit directly underneath the prey [11]. The newly hatched larvae often penetrate into the oocysts of *Icerya* and their second- and third-instar nymphs to feed under the abdomen [12]. As a native species, *N. pumilus* is well adapted to the local environment in China, and there is no potential risk of invasion. Therefore, *N. pumilus* exhibits huge advantages in controlling *Icerya* pests in China. However, the relevant molecular mechanisms underlying the adaptation of *N. pumilus* to the prey *Icerya* are still unclear.

The adaptations to a very large range of food sources and the acquisition of multiple nutritional niches marks the remarkable evolutionary success of insects [13]. Previous studies have shown that the physiological and biochemical processes of chemosensing, digestion and detoxification could largely affect the feeding range and diet adaptation processes of insects [14–22]. For instant, the gene families that encode odorant-binding proteins and odorant receptors (ORs) act important roles in the lifestyle of insect species, allowing them to locate new sources of diet [23, 24]. In addition, diet is also found to be able to shape insect immunity [13, 25], and insects have developed an immune system to adapt to the changes in diet and microbiota in different environments [26]. Accordingly, a comprehensive investigation to genomic and transcriptomic features would be helpful for exploring the mechanisms that might be involved in prey adaptation of *Novius*.

In this study, we present a high-quality draft genome of *N. pumilus.* Through comparative and evolutionary analyses with genomes of other four ladybirds, we aimed to investigate the genomic basis underlying the feeding habits of *N. pumilus.* Moreover, we further examined the differences in transcriptional regulations between feeding and not feeding treatments of larvae and adults of *N. pumilus*, respectively, to explore the mechanism of the feeding habit and adaptation of *N. pumilus* to *Icerya*. Our findings would shed light on the utilization of *Novius* in biocontrol.

## Results

### General genomic features of *N. pumilus*

A total of 48.82 gigabase pairs (Gb) of raw data, where 45.36 Gb are of high quality (clean reads), were generated with PromethION DNA sequencing (Oxford Nanopore, UK). Furthermore, we sequenced 128 Gb additional data using Illumina platform. The Nanopore data were first assembled using Wtdbg v 2.2 [27], followed by polishing with Racon v1.32 [28] and Pilon v1.21 [29], which produced a 176.16 Mb genome assembly with 942 contigs with a Contig N50 of 7.58 Mb and L50 of 8 in this study (Table 1). The genome of *N. pumilus* has the smallest Contig L50 and relatively large N50 compared to those of the other 13 Coleoptera genomes.

**Table 1:** The basic information of the 14 Coleoptera species genomes used in this study

Species	Family	Genome size (Mb)	Contigs	ContigN50 (bp)	Contig L50	Scaffold N50 (bp)	Scaffold L50	Reference
*Agrilus planipennis* Fairmaire, 1888	Buprestidae	353.06	23,186	28,329	3132	1,113,421	91	[30]
*Photinus pyralis* (Linnaeus, 1767)	Lampyridae	471.51	7909	170,308	757	47,017,841	5	[31]
*Nicrophorus vespilloides* Herbst, 1783	Staphylinidae	195.27	8129	67,958	623	122,407	344	[32]
*Onthophagus taurus* (Schreber, 1759)	Scarabaeidae	267.08	10,620	101,202	519	337,157	160	[30]
***Novius pumilus*** **(Weise, 1892)**	Coccinellidae	176.16	942	7,579,481	8	–	–	This study
*Cryptolaemus montrouzieri* Mulsant, 1850	Coccinellidae	988.11	398	10,375,940	26	–	–	[13]
*Harmonia axyridis* (Pallas, 1773)	Coccinellidae	423	2785	1,254,346	79	2,054,022	44	[33]
*Coccinella septempunctata* Linnaeus, 1758	Coccinellidae	556.17	66,468	21,314	5248	67,752	709	[34]
*Propylea japonica* (Thunberg)	Coccinellidae	803.93	4778	813,984	177	100,336,366	4	[35]
*Tribolium castaneum* (Herbst, 1797)	Tenebrionidae	204	7059	73,049	512	4,456,720	12	[36]
*Aethina tumida* Murray, 1867	Nitidulidae	234	3063	298,879	193	–	–	[37]
*Dendroctonus ponderosae* (Hopkins, 1902)	Curculionidae	252.85	40,744	10,101	5474	465,763	137	[38]
*Anoplophora glabripennis* (Motschulsky, 1854)	Cerambycidae	706.95	26,749	80,490	2230	678,234	269	[19]
*Leptinotarsa decemlineata* Say, 1824	Chrysomelidae	641.99	45,556	46,596	3600	139,046	1179	[18]

In total, about 96.39% reads and 92.05% paired-end reads were mapped to the assembled genome. The coverage of the Nanopore and Illumina data is 242× and 640×, respectively. Besides, the application of the Benchmarking Universal Single-Copy Orthologs (BUSCO, Insecta set) pipeline [39] showed that the *N. pumilus* genome compared well with the other insect genomes in the OrthoDB v10.1 database [40], in terms of completeness. We found approximately 1337 orthologous complete genes (C: 97.8%; including 1305 orthologous complete genes and single-copy genes (S: 95.5%) and 32 orthologous complete genes and duplicates (D: 2.3%)), 7 orthologous fragmented genes (F: 0.5%) and 23 missing genes (M: 1.7%) (Additional file 1: Table S1), indicating that the genome was of good quality.

### Genome annotation

**Fig. 1:**
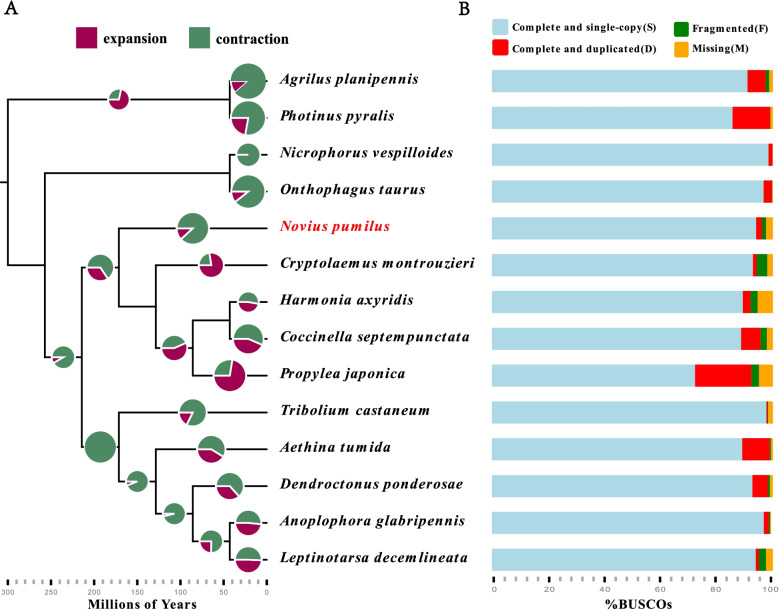
Evolution of genes families and estimated completeness of the gene sets of 14 insect species. **A** The species’ ultrametric tree was adapted from Mckenna et al. [20]. We used CAFE (Computational Analysis of gene Family Evolution) to infer the size change of gene families. This summary tree shows the average expansion/contraction (radius of node circles), where for each circle, purple highlights the percentage of expanded gene families, and green highlights the percentage of contracted gene families. **B** Completeness of offcial gene sets of each insect species were assessed by applying the Benchmarking Universal Single-Copy Orthologs (BUSCO, Insecta set) pipeline

Annotation of the *N. pumilus* genome was carried out using FunAnnotate v1.8.1 [41], and yielded a final set of 15,772 genes and 17,195 protein sequences. Application of the BUSCO pipeline showed that this gene set had 96.1% complete genes (94.1% single-copy genes and 2% duplicates), 1.5% fragmented genes and 2.4% missing genes at the protein level in the Insecta set of OrthoDB (Fig. 1B; Additional file 1: Table S1). In the functional annotation of this protein set, 14,691 (85.4%) were found in the National Center for Biotechnology Information (NCBI) nonredundant (NR) subset, 10,866 (63.2%) in Swiss-Prot/UniProt, 11,126 (64.7%) in at least one protein domain in Pfam, 11,269 (65.5%) in the Gene Ontology (GO) database, and 5071 (29.5%) in the Kyoto Encyclopedia of Genes and Genomes (KEGG) pathway database [42] (Additional file 1: Table S2).

### Gene content comparison

We identified 21,017 orthogroups (OGs) using OrthoFinder v2.4.0 [43] among *N. pumilus* and the other 13 coleopteran insects included in our analyses (Table 1). Of them, 5818 OGs (7038 *N. pumilus* genes) were present in all coleopteran insects analyzed in this study. A total of 14,283 OGs in *N. pumilus* clustered with the other beetle species, and 281 OGs were found to be unique to *N. pumilus*, consisting of 1382 genes (Additional file 2: Table S1). GO enrichment analysis revealed that these unique OGs were enriched in cysteine-type peptidase activity and serine-type endopeptidase activity (false discovery rate (FDR) < 0.05).

### Gene selection and gene family evolution in *N. pumilus*

To access the genomic basis underlying the *N. pumilus*-specific adaptation to *Icerya*, we performed genome-wide comparative analyses with other four ladybirds and nine outgroup beetles to identify the gene families under expansion/contraction, as well as the positively selected genes, in the genome of *N. pumilus*.

Firstly, a positive selection analysis based on the single-copy orthologous genes was performed on the genome of *N. pumilus* by aligning coding sequences (CDSs) from each OG to their orthologs in the 13 other beetles using branch-site model detection in CodeML [44] (Table 1). A total of 711 codon alignments were generated from 971 single-copy genes, while the others were excluded due to codon-to-protein inconsistency, which might have been caused by the different gene predictions in the database. The branch-site model showed that 88 genes were under significant positive selection pressure (likelihood ratio test (LRT), *P* < 0.05) among the 711 single-copy genes (Additional file 2: Table S2). Of them, four encode leucine rich repeats (LRRs) related to immunity and one encodes glycosyltransferase related to digestion.

Next, we investigated the expansions and contractions of OGs in the *N. pumilus* genome. As revealed by the clustering algorithm implemented in CAFE v4.2.1 [45], we found that the number of contracted OGs was far greater than that of expanded OGs in the *N. pumilus* genome (8221 and 1103, respectively). A total of 17 OGs underwent significant expansion (*P* < 0.05), where one encoded chemosensor (specifically, an OR), two encoded digestive enzymes (both of which were papain family cysteine proteases), and two encoded antimicrobial enzymes (namely, an LRR and a gamma interferon inducible lysosomal thiol reductase (GILT)) (Table 2; details are provided in Additional file 2: Table S1). Of the 14 significantlycontracted OGs, one encodes cysteine-rich secretory protein (CRISP) related to digestion (Table 2).

**Table 2:** The number of gene copies that OGs expanded /contracted in *N. pumilus* than the last common ancestor of ladybirds

Annotation	+/−	***P*** - value	Pfam	n^**a**^	In L Null model	Alternative model	Easycodeml model	2Δl^**c**^(df = 1)	***P -*** value
Papain family cysteine protease	+ 10	0.0293442	PF00112	(130) 25	−81,374.615334	−81,370.385291	Model A vs. Model A null	8.460086	0.003630247^*^
GIY-YIG catalytic domain	+ 10	0.0115812	PF01541	(77) 20	−63,449.8	−63,449.8	Model A vs. Model A null	2.00E-06	0.997743
Zinc finger, C2H2 type	+ 65	2.6335E-23	PF00096	(78) 71	−76,378.4	−76,373.7	Model A vs. Model A null	9.333846	0.002249597^*^
Papain family cysteine protease	+ 11	0.00067968	PF00112	(62) 16	−42,889.4	−42,889.1	Model A vs. Model A null	0.437724	0.508223
Bacterial extracellular solute-binding proteins, family 3	+ 55	6.5167E-21	PF00497	(60) 59	−65,378.7	−65,372.3	Model A vs. Model A null	12.70093	0.000365475^**^
Histidine phosphatase superfamily (branch 2)	+ 9	0.00432895	PF00328	(59) 15	−50,522.7	−50,522.7	Model A vs. Model A null	0	1
Retroviral aspartyl protease	+ 49	2.2232E-19	PF00077	52	31,763.07136	31,763.07136	M1a vs. M2a	0	1
YqaJ-like viral recombinase domain	+ 6	0.0286801	PF09588	(46) 11	−20,486.4	−20,481.5	Model A vs. Model A null	9.742052	0.001800997^*^
CRAL/TRIO domain	+ 10	0.00223833	PF00650	(47) 16	−32,465.4	−32,462.9	Model A vs. Model A null	5.072066	0.024314388^*^
unknown	+ 43	5.7991E-17	unknown	46	−39,594.10792	−39,394.06476	M1a vs. M2a	400.0863	0^**^
7tm Odorant receptor	+ 14	3.346E-05	PF02949	(39) 18	−28,871.9	−28,867.7	Model A vs. Model A null	8.308994	0.003944924^*^
Leucine rich repeat	+ 36	4.71E-15	PF13855	38	31,494.14597	31,494.14597	M1a vs. M2a	0	1
unknown	+ 34	3.0939E-14	unknown	36	− 3339.182609	− 3330.176841	M1a vs. M2a	18.01154	0.0001227^*^
Integrase core domain	+ 6	0.0286801	PF00665	(35) 11	−22,990.3	−22,990.3	Model A vs. Model A null	0	1
unknown	+ 22	2.2991E-09	unknown	(25) 24	− 8370.951276	− 8369.324548	Model A vs. Model A null	3.253456	0.071273
Domain of unknown function (DUF4769)	+ 3	0.0234649	PF15992	(23) 4	−11,711	− 11,707.8	Model A vs. Model A null	6.345562	0.011767532^*^
Gamma interferon inducible lysosomal thiol reductase (GILT)	+ 18	1.1647E-08	PF03227	19	− 8954.59711	− 8930.826609	M1a vs. M2a	47.541	0^**^
Core histone H2A/H2B/H3/H4	−5	0.0475987	PF00125	(89) 1	− 8236.68	− 8196.31	Model A vs. Model A null	−80.7418	0^**^
Baculovirus FP protein	−6	0.00719925	PF03258	(87) 0	−44,251.4	−44,246.9	Model A vs. Model A null	−8.99553	0.002706408^*^
Matrixin	−4	0.047769	PF00413	(80) 0	−34,306.30303	−34,295.78115	Model A vs. Model A null	21.04375	0.000004489^*^
Core histone H2A/H2B/H3/H4	−5	0.0475987	PF00125	(77) 1	− 9959.15	− 9959.15	Model A vs. Model A null	2.00E-06	0.998872
unknown	−5	0.0183516	unknown	(73) 0	−27,707.9	− 27,682.2	Model A vs. Model A null	51.30864	0^**^
Core histone H2A/H2B/H3/H4	−4	0.047769	PF00125	(66) 0	− 4648.924631	− 4617.070009	Model A vs. Model A null	63.7092439999997	0^**^
DDE superfamily endonuclease	−4	0.047769	PF13359	(58)0	−17,817.03628	−17,815.55146	M1a vs. M2a	2.96964	0.226543
RNase H	−4	0.047769	PF00075	(51) 0	−38,944.3	−38,937.9	Model A vs. Model A null	12.86602	0.000334602^**^
Retrotransposon gag protein	−4	0.047769	PF03732	(49) 0	−16,647	−16,636.2	Model A vs. Model A null	21.48437	0.000003567^**^
unknown	−4	0.047769	unknown	(46) 0	−10,117.57741	−10,103.67858	M1a vs. M2a	27.79766	0^**^
Transposase protein	−4	0.047769	PF12017	(44) 0	−28,053	−28,048.2	Model A vs. Model A null	9.461622	0.002098155^*^
Cysteine-rich secretory protein family	−4	0.047769	PF00188	(34) 0	−50,366.08197	−50,366.08197	Model A vs. Model A null	0	1

^a^Number of sequences in the data set

^**^Signifcant at the 0.1% level

^*^Signifcant at the 5% level

**Fig. 2:**
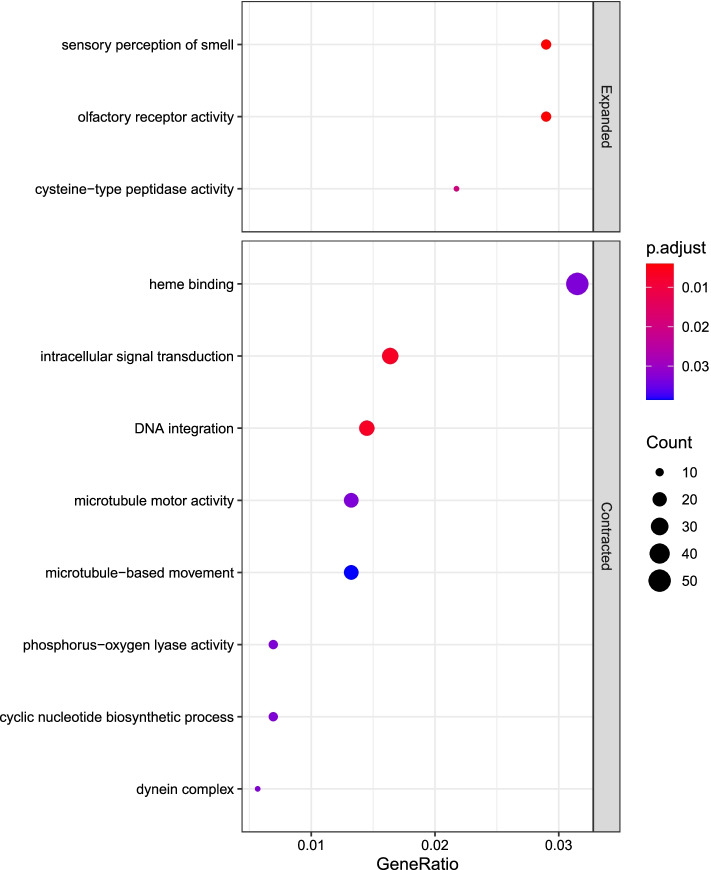
The GO enrichment of the expanded and contracted families of *N. pumilus* genome compared to other 13 beetles. The x axis ‘GeneRatio’ represents the ratio of the DEGs falling in a certain term to the total number of genes annotated in the same term. The y axis is the significant GO terms identified in our analysis. The significance level (adjusted *p*-value) is quantified by the color of circle where more red represents more significance (smaller adjusted *p*-value), while more blue represents less significance (larger adjusted *p*-value)

The GO enrichment results showed that the enriched expanded OGs were associated with olfactory receptor activity, sensory perception of smell related to olfaction and cysteine-type peptidase activity related to digestion (Fig. 2). The significantly enriched contracted OGs were mainly associated with heme binding, DNA integration and intracellular signal transduction (Fig. 2).

Then, we conducted selective pressure analysis for the OGs with significant expansion or contraction in CAFE (*P* < 0.05) to gain a better understanding of the mechanisms of prey adaptation. The results showed that among the 17 OGs with significant expansion and the 14 OGs with significant contraction, 10 and 8 OGs were subjected to significant positive selection (*P* < 0.05), respectively (Table 2), as determined by the command Branch Site Model or Site Model in EasyCodeML [46].

### Identification and functional analysis of differentially expressed genes

To clarify the genes that play important roles when *N. pumilus* feeding the cottony cushion scale and investigate the mechanisms underlying its feeding and adaptation to natural preys *Icery*a, we performed comparative transcriptome analysis between *N. pumilus* individuals feeding or not feeding on *I. aegyptiaca*. A fold change > 2 or < 0.5 and FDR Q-value < 0.05 were used as the criteria for defining differentially expressed genes (DEGs) in adults and larvae between feeding and not feeding on *I. aegyptiaca*. In total, 1322 DEGs were identified, including 831 downregulated genes and 491 upregulated genes, in the larvae feeding on *I. aegyptiaca* compared to those without feeding. For convenience, DEGs were labeled “upregulated” or “downregulated” if they were higher or lower expressed when feeding on *I. aegyptiaca* than not feeding. The fold changes (log2 ratios) in the DEGs ranged from − 27.82 to 10.56 (Additional file 3: Table S1). We also detected the DEGs in *N. pumilus* adults between feeding and not feeding on *I. aegyptiaca*. Compared with the larvae, there were fewer DEGs (91) in the adults, where 40 were upregulated, and 51 were downregulated. The fold changes (log2 ratios) of the DEGs ranged from − 7.73 to 23.61 (Additional file 3: Table S2).

**Fig. 3:**
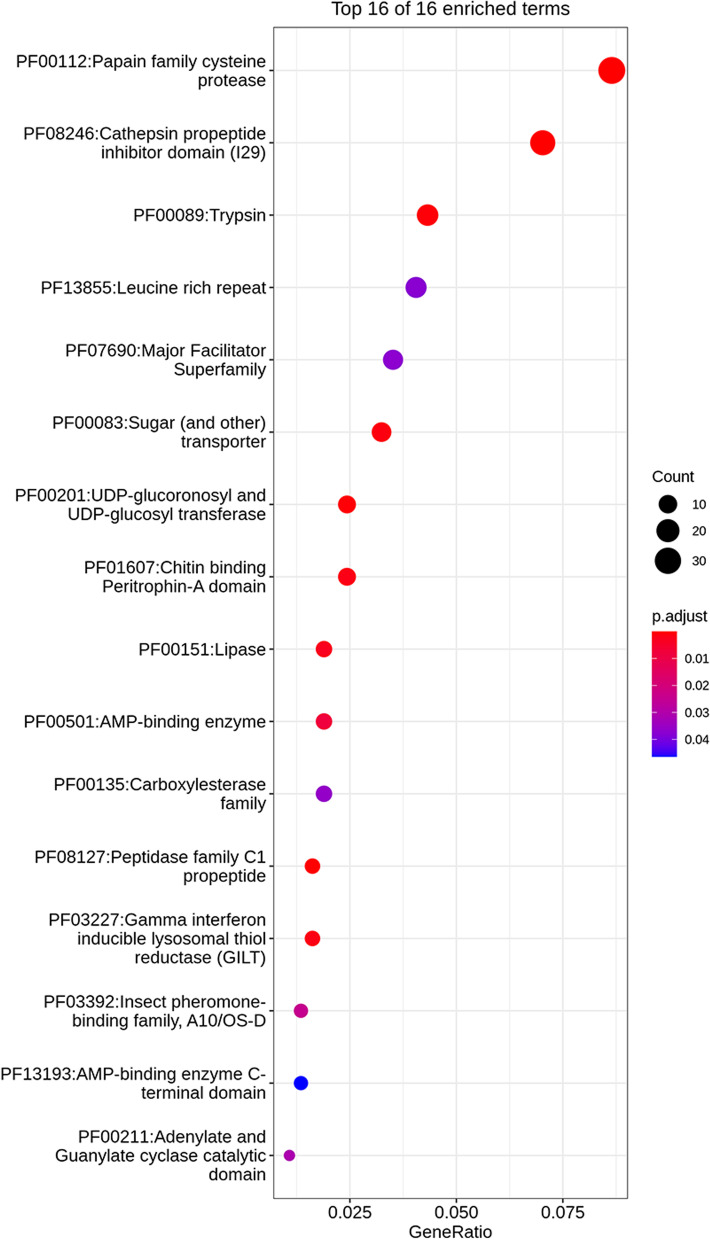
Function of the studied genes, their accession in Pfam database and result of enrichment analysis of their upregulated DEGs of *N. pumilus* larvae in feeding on *I. aegyptiaca* and not feeding. Terms with adjusted *p*-value < 0.05 were considered as significantly enriched. The x axis ‘GeneRatio’ represents the ratio of the DEGs falling in a certain term to the total number of genes annotated in the same term. The y axis is the significant Pfam terms identified in our analysis. The significance level (adjusted *p*-value) is quantified by the color of circle where more red represents more significance (smaller adjusted *p*-value), while more blue represents less significance (larger adjusted* p*-value)

Then, we performed Pfam enrichment analyses on the DEGs between the two conditions (feeding vs not feeding) in the larvae and adults, respectively. Sixteen Pfam domain terms were identified to be significantly enriched for the upregulated genes in the larvae (Q-value < 0.05), which were mainly related to development, energy metabolism, chemosensing, digestion, detoxification and immunity (Fig. 3). These enriched domain terms included papain family cysteine proteases, cathepsin propeptide inhibitor domain (I29), chitin binding peritrophin-A domain, UDP-glycosyltransferase (UGT), trypsin, GILT, carboxylesterase (COE), LRR proteins, and insect pheromone-binding family (insect PBP). For the adults, the upregulated genes were mainly enriched in LRR, while transcription factors were overrepresented for downregulated genes (Fig. S1 and S2).

In the significantly expanded OGs, genes were found to be overexpressed in the larvae when feeding on *I. aegyptiaca*. Among them, 11 of 38 LRR and four of 19 GILT genes were significantly upregulated, with some of the log2-fold change values greater than four (i.e., the genes were 16 times more abundant than that they were not feeding; Additional file 3: Table S2). Nine of 16 and six of 25 genes in the two digestion-related homologous gene families of papain cysteine proteases were also significantly upregulated, and some of the log2-fold change values were greater than nine (i.e., the genes were 512 times more abundant than those not feeding on *I. aegyptiaca*; Additional file 3: Table S2). Comparatively, the adults had fewer significant DEGs in these significantly expanded OGs when feeding on *I. aegyptiaca*, where only one LRR gene was upregulated and one GILT gene was downregulated.

**Fig. 4:**
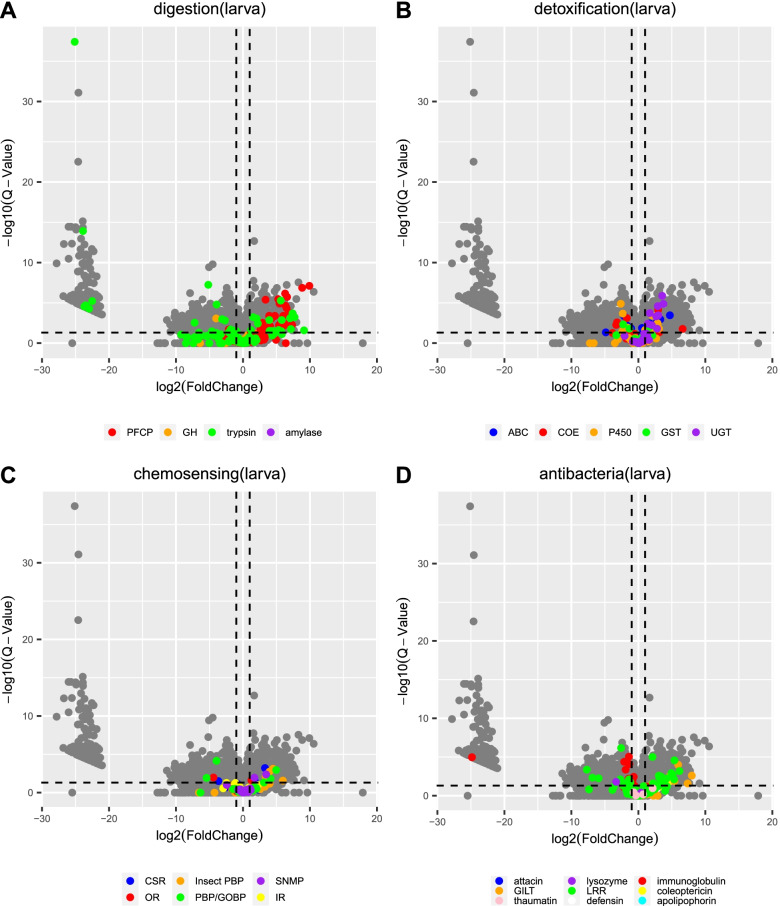
Analyses of DEGs related to **A** digestion, **B** detoxification, **C** chemosensor, **D** antibacterial and immunity, when the larvae feeding on *I. aegyptiaca* compared with the no feeding set. DEGs were identified using volcano plots. Horizontal coordinate: log2(fold change), vertical coordinate: -log10(Q-value). The studied genes were coloured, and those DEGs were marked as solid circle. Information of Pfam accession of genes can be found in Additional file 1: Table S2

In both larvae and adults, we also examined the expression levels of all genes from the gene families of digestion, detoxification, chemosensing and immunity, according to Pfam annotations, between feeding and not feeding on *I. aegyptiaca*. In the larvae, we identified 94 DEGs associated with digestion-related genes when feeding on *I. aegyptiaca*, where 32 DEGs encode trypsin, 27 encode cathepsin propeptide inhibitor I29 proteins, 33 encode papain family cysteine proteases, one encodes a GH family protein 18 and one encodes an alpha-amylase. Most of these genes, for example, 32/33 of the papain family cysteine protease and 16/32 of the trypsin genes were upregulated (Fig. 4A).

For the detoxification gene family, genes encoding P450s, UGTs, COEs, ATP-binding cassette (ABC) transporters, and glutathione S-transferases (GSTs) were differentially expressed (Fig. 4B) in the larvae feeding on *I. aegyptiaca*. Among these genes, seven P450, nine UGT, seven COE, five ABC transporter and one GST genes were upregulated (Fig. 4B). Additionally, we identified five genes encoding OBPs/GOBPs, five encoding chemosensory proteins (CSPs), three encoding chemosensory receptors and two encoding ORs that were significantly differentially expressed in the larvae feeding on *I. aegyptiaca* (Fig. 4C). Among these chemosensory DEGs, most were upregulated. And all of the CSP and SNMP genes were upregulated. In terms of antibacterial activity or immunity, we found that one gene encoding lysozyme, one gene encoding coleoptericin, six genes encoding GILTs, 16 genes encoding immunoglobulin and 26 genes encoding LRR proteins were differentially expressed when the larvae feeding on *I. aegyptiaca* compared with not feeding (Fig. 4D). It is worth noting that among the DEGs, all GILT and coleoptericin genes were upregulated, while all immunoglobulin and lysozyme genes were downregulated, during the larvae feeding on *I. aegyptiaca* (Fig. 4D).

**Fig. 5:**
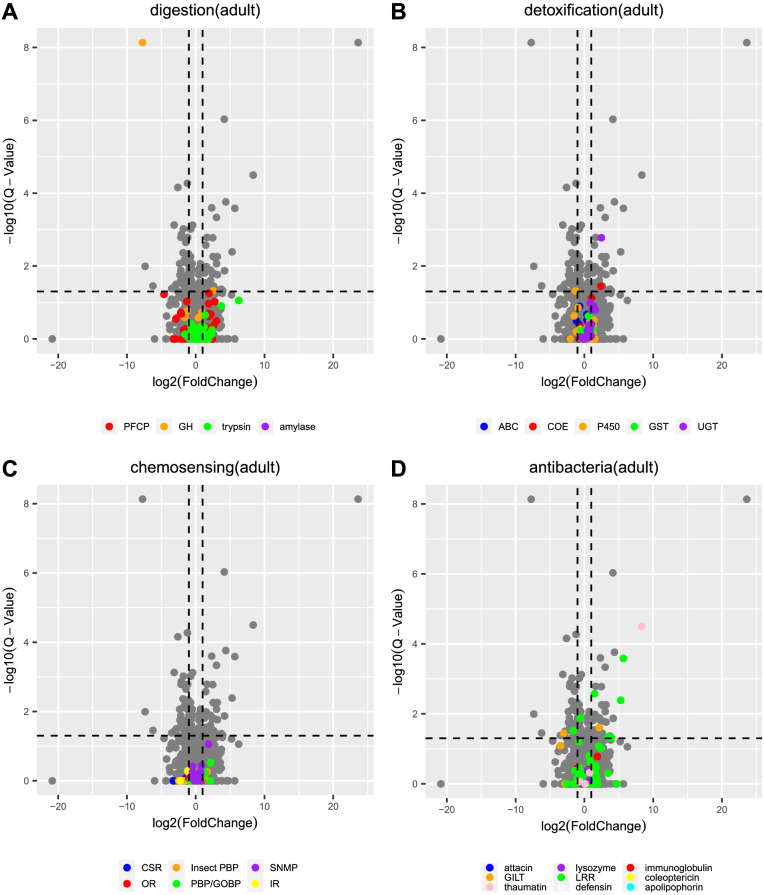
Analyses of DEGs related to **A** digestion, **B** detoxification, **C** chemosensor, **D** antibacterial and immunity, when the adult feeding on *I. aegyptiaca* compared with the no feeding set. DEGs were identified using volcano plots. Horizontal coordinate: log2(fold change), vertical coordinate: -log10(Q-value). The studied genes were coloured, and those DEGs were marked as solid circle. Information of Pfam accession of genes can be found in Additional file 1: Table S2

For the *N. pumilus* adults feeding on *I. aegyptiaca*, two genes related to digestion were differentially expressed (Fig. 5A), including one downregulated and one upregulated gene both from GH family 1 (Fig. 5A). Three detoxification-related DEGs were identified, including one downregulated P450 gene, and two upregulated genes encoding COE and one UGT, respectively (Fig. 5B). There were no DEGs among the chemosensory-related genes (Fig. 5C). Among the immunity-related DEGs, six LRR genes were differentially expressed, where five were upregulated (Fig. 5C). In addition, one GILT gene and one thaumatin gene were also upregulated (Fig. 5D).

## Discussion

In the past 20 years, more than 100 insect genomes have been sequenced [47], which largely expand our understanding of the biodiversity of insect habits, behaviors, and long-term evolutionary relationships [48]. However, there are still many species that play important roles in agriculture deserving further study. In this study, we report the first draft genome of *N. pumilus*, which is of good continuity and high completeness (Additional file 1: Table S1). The size of *N. pumilus* genome was smaller than the 13 other Coleoptera genomes included in our study (Table 1). Comparative evolutionary analysis showed that several OGs related to chemosensor, digestion, and immunity had undergone significant expansion in the *N. pumilus* genome, and some genes of these OGs were also favored by positive selection (Table 2). With the help of our new completely sequenced *N. pumilus* genome, gene regulation induced by feeding on *I. aegyptiaca* was explored. Compared to not feeding, DEGs when feeding on the natural prey *I. aegyptiaca* were mainly enriched for development, energy metabolism, chemosensing, digestion/detoxification and immunity, which were similar to the results of OGs expansion and positive selection analyses. These genes are usually involved in the dietary adaptation of both phytophagous and predatory beetles [13], indicating that they may also play important roles in the prey adaptation of *N. pumilus*.

Insects are always harassed by a wide array of microbial challenges, e.g., pathogenic bacteria, from diets, hence, insects have evolved a strong immune system to cope with the infections [26, 49, 50]. The LRR serves as an important protein binding target in innate [51], and GILT mediates the degradation of endocytosed proteins and alters the immune response characters [52]. In *N. pumilus*, we found that LRR and GILT gene families were significantly expanded, and the genes from GILT family was also favored by positive selection. At the same time, LRR and GILT genes were found to be significantly upregulated in both adults and larvae feeding on *I. aegyptiaca*. The enhanced immune process may be due to the adverse impacts induced by the microbial on the surface or in the gut of *Icerya*, which is the so-called “bacterial challenges”. Similarly, in the insect *Monochamus saltuarius*, these two antimicrobial proteins were reported to play important roles in responding to bacterial challenges [17]. Thus, the above results indicate that LRR and GILT may play crucial roles in promoting the adaptation of *N. pumilus* to the preys. In contrast, *C. montrouzieri* employs different pathways to cope with the nature prey mealybugs, for example, via enhancing immune effector genes [13]. These differences between *N. pumilus and C. montrouzieri* may reflect the species specificity in immune response mechanisms to the prey of ladybirds.

It is interesting to mention that genes related to digestion and detoxification, which could help to cope with toxic challenges from preys [53–60], were significantly upregulated in the larvae of *N. pumilus* when feeding on *I. aegyptiaca* compared with not feeding, while there were only few significant DEGs in adults. They, as well as chemosensory genes, likely influence insect behaviors such as searching for food, preference for food, oviposition sites, and mates [61–63]. These differences suggest that though both the adults and larvae of *N. pumilus* obligately feed on *Icerya*, they might employ different mechanisms for handling the stress from the prey. Further studies such as electrophysiological and chemical experiments are required to confirm this hypothesis.

Massive expansion of natural enemy insects can guarantee efficient biological control of pests. The availability of a cost-effective natural prey or artificial diet is the key to the successful large-scale production of natural arthropod enemies for biological control [64]. *Novius* species are highly oligophagous and have important application value in biological control. At present, large-scale reproduction of ladybirds in this genus still requires large-scale breeding of their natural prey, which is costly and time consuming. In this study, with the help of our new completely sequenced *N. pumilus* genome, we explored the expanded or contracted OGs and the gene regulations potentially associated with the feeding habits and adaptation to the natural prey *Icerya*, which provides novel insights into the underlying mechanisms of diet development, as well as speciation.

## Conclusions

The high-quality whole genome sequence of *N. pumilus* reported here provide more insights into the evolutionary interactions between *Novius* and their preys *Icerya*. The OGs involved in feeding habits and adaptions, such as chemosensing, digestion and immunity, were significantly expanded, and some genes in the OGs were favored by positive selection. Consistently, these functions were also enhanced when feeding on the nature prey *I. aegyptiaca*. In addition, the processes of chemosensing, digestion and detoxification were more activated in the larvae when feeding than in the adults, suggesting that the mechanism responding to the prey stress may be different between mature and unmature *N. pumilus* individuals. These novel findings enriched the current knowledge on molecular mechanisms of prey adaptation in *N. pumilus* and represent a valuable resource for future application of *N. pumilus* and other *Novius* species in pest biocontrol. Moreover, deeper studies on prey adaption of *Novius* species can be operable with the help of our genome.

## Methods

### DNA extraction, genome sequencing and assembly

DNA was extracted from the whole bodies of 21 female adults of *N. pumilus.* These individuals were wild caught from the *Magnolia denudata* tree in South China Agricultural University, Guangzhou, China. Genomic DNA was extracted using the CTAB method [65]. The quality and concentration of the extracted genomic DNA was measured by 1% agarose gel electrophoresis and a Qubit fluorimeter (Invitrogen, Carlsbad, CA, USA). High-quality DNA was used for subsequent Nanopore and Illumina sequencing.

Approximately 12 μg of genomic DNA was used for preparing Nanopore long-read library. Briefly, genomic DNA was first randomly fragmented, and DNA damage and end were repaired. Sequencing adapters were then ligated to fragmented DNA. The library was sequenced on a PromethION DNA sequencer (Oxford Nanopore, Oxford, UK). The raw data were then filtered to remove short sequence reads (< 5 kilobase pairs (kb)) and reads with low-quality bases (Q30 < 90%) using Nanofilt v2.3.0 [66]. To assemble Nanopore sequencing data, Canu v1.5 [67] was implemented to generate more accurate self-corrected reads with a corrected error rate of 0.05. Wtdbg v 2.2 [27] was employed for assembly with default settings. Racon v1.32 [28] was performed to correct the assembly with Nanopore reads through two rounds with default settings. And genomic DNA was also sequenced on the Illumina HiSeq X Ten platform (Illumina, San Diego, CA, USA) for further error correction. The Illumina sequenced data were filtered to remove reads with low-quality bases and adapters through Trimmomatic v0.36 [68] with default settings. Pilon v1.21 [29] was applied to correct the Nanopore assembly with Illumina reads through three rounds with default settings.

Primary genome assemblies may contain contamination, such as sequences from symbiotic bacteria, which should be removed. To avoid contamination, we searched the NCBI nucleotide sequence (NT) database (downloaded from https://ftp.ncbi.nlm.nih.gov/blast/db) by BLAST v2.8.1+ [69] with a E-value cutoff of 10^− 5^ and then manually checked the matched hits of the contigs. Forty-two contigs were identified to be from bacteria, which accounts for less 3% of the total sequences (4.83 Mb out of 187.59 Mb). And these bacteria were mainly intestinal symbiotic bacteria of *N. pumilus* (unpublished results). To ensure the accuracy, these contigs of bacterial origin were filtered out from our downstream analysis.

### Genome refinement, gene prediction and functional annotation

Genome refinement, gene prediction and functional annotation of 5 ladybirds were carried out mainly using FunAnnotate v1.8.1 [41], a gene prediction pipeline, combined with some additional procedures (Additional file 4: Fig. 3). First, the duplicated contigs (27 contigs accounting for 0.33 Mb) were removed by the “clean” module in the FunAnnotate pipeline, which used Minimap2 [70] to align the shorter contigs to the longer contigs one by one and removed the shorter contigs if the percent identity and the percent coverage of the alignments were both above 95. Contigs with less than 500 bases were also excluded. All contigs were then sorted by length and renamed to prepare downstream gene predictions. The repetitive elements of the prepared genome were identified and masked through the FunAnnotate “mask” module. Repetitive elements were predicted by RepeatModeler v1.0.11 [71], followed by soft masking with RepeatMasker v4.0.9 [72]. The RNA-Seq data of each species in different life stages and under different treatments (Additional file 3: Table S3) were mapped using HISAT2 v2.2.0 [73] and were used to generate a genome-guided assembly by Trinity v2.8.5 [74] and StringTie v2.1.4 [75]. Subsequently, PASA v2.4.1 [76] assembly and prediction were performed with Minimap2, GMAP v2019-03-15 [77] and BLAT v36x2 [78] as aligners through the FunAnnotate “train” module, setting the maximum intron length to 20,000 bp, because more than 99.5% of the introns identified in the assembly transcriptome from our previous study are less than 20,000 bp [79]. Gene models were generated by the consensus of various prediction methods through the FunAnnotate “predict” module. The ab initio gene predictions were conducted by SNAP v2006-07-28 [80], AUGUSTUS v3.3.3 [81], GlimmerHMM v3.0.4 [82], GeneMark-ET v4.46 [83] and the ET mode of BRAKER v2.1.1 [84]. HISAT2 RNA-seq alignments were used to train GeneMark-ET and AUGUSTUS, followed by AUGUSTUS optimization. Minimap2, GMAP and BLAT spliced alignments generated in the PASA assembly above were used as transcript evidence. The protein evidence was obtained by Exonerate v2.4.0 [85] alignment after a DIAMOND v2.0.4 [86] search against a customized protein database containing all the proteins in UniProtKB/Swiss-Prot [87] and proteins of the species under Arthropoda in the NR database. Additionally, a high-quality GMAP “gff3_gene” prediction was conducted using complete coding sequence (CDS) from TransDecoder v5.5.0 [88] prediction of the genome-guided transcript assembly obtained above, with identity> = 95%, coverage = 100% and completely consistent CDS regions. Together with the PASA/TransDecoder prediction, all these predictions were submitted to EVidenceModeler (EVM) v1.1.1 [89] to obtain the final gene models. Repeats were used in EVM consensus model building, the maximum intron length was set as 20,000, and the long introns with more than 500 bp were searched again to find the nested genes. The predictions and their weights used in EVM are listed in Additional file 3: Table S4. PASA was used to capture untranslated regions (UTRs) and refine gene models through the FunAnnotate “update” module. Only alternative transcripts with more than 10% expression in Kallisto v0.46.0 [90] pseudoalignments compared with the highest transcripts were retained. The problematic gene models were fixed in the FunAnnotate “fix” module.

Then, the predicted proteins were annotated using the FunAnnotate “annotate” module. Domains within the proteins were searched against the Pfam v33.1 [91] database using HMMER v3.3 [92] and the InterPro v71.0 [93] database using InterProScan v5.32 [94]. EggNog and COG annotations were obtained through Eggnog-mapper v2.0.0 (EggNog v5.0) [95, 96]. By combining the results of InterProScan and Eggnog-mapper, we also obtained GO and KEGG pathway annotations. DIAMOND was used to search UniProtKB/Swiss-Prot, and then, the names and product names of the proteins were identified by combination of the UniProtKB/Swiss-Prot and Eggnog-mapper results. In addition, BUSCO annotations with the Endopterygota set of OrthoDB v9 [97], CAZy [98] annotations by HMMER and MEROPS [99] annotations by DIAMOND were generated. The secreted signal peptides and the transmembrane structures were predicted by SignalP v5.0 [100] and Phobius v1.01 [101]. In addition to the above FunAnnotate annotations, we predicted the mitochondrial transit peptides by TargetP v2.0 [102]. The cutoff for all the E-values above was 1e-5. We provide the flowchart of the FunAnnotate annotation in Additional file 4: Fig. 3.

### Orthology search and gene family evolution

We selected 14 beetle species with different feeding habits for orthologous analysis. Among these species, four are ladybird species of the Coccinellidae family (*Coccinella septempunctata*, *Propylea japonica*, *Harmonia axyridis* and *Cryptolaemus montrouzieri*) with available genome sequences, and they exhibit apparently a relatively wider prey range than *N. pumilus*. These four ladybirds can feed on aphids, mealybugs and whiteflies [1], while *N. pumilus* mainly feeds on the pests of the *Icerya* [103]. There have been several genomes of *H. axyridis* sequenced, among which we only selected a chromosome-level genome from Chen et al. (2021) [33] to use in the analysis. And we selected nine representative outgroup species from different families of Coleoptera, which have clear phylogenetic status according to previous studies. Furthermore, the quality of the genome sequencing of these species are high, which are reliable for the analysis. The gene sets of the 14 Coleoptera genomes predicted from RNA-Seq data were used (Table 1) to identify orthologous genes. Then, we used the longest isoforms of the protein sequences of the 14 species as input for OrthoFinder v2.4.0 [43] to identify orthologous genes with default settings. Protein domains within genes were searched against the Pfam v32 database using InterProScan v5.32 with a cutoff E-value of 10^− 5^. Information on the protein domains was subsequently assigned to the orthogroups using KinFin [104]. Furthermore, these orthogroups were used as input for CAFE v4.2.1 [45] to assess gene family contraction and expansion dynamics using the birth/death parameter (λ). The species tree (Fig. 1A) used in CAFE was adapted from a recently published Coleoptera phylogeny [20]. In each branch, OGs with *p*-values < 0.05 were considered significant expansions or contractions. Furthermore, we performed GO enrichment analysis for the expanded and contracted orthogroups in *N. pumilus*, using the R package ClusterProfiler [105]. Where the significance level (*p*-value) is assessed based on hypergeometric distribution, and the FDR in multiple comparison is further controlled using an adjusted *p*-value of Benjamini-Hochberg Procedure with the cutoff of 0.05.

### Tests of positive selection

We used the branch-site model (parameters: null hypothesis: model = 2, NSsites = 2, fix_omega = 1, omega = 1; alternative hypothesis: model = 2, NSsites = 2, fix_omega = 0, omega = 1) in PAML v4.8a [106] to identify the genes with positively selected sites in the *N. pumilus* genome for single-copy ortholog sequences, using the foreground branches in the species tree (Fig. 1A) and labeling at the *N. pumilus* node. Then, LRTs were performed to detect positive selection on the foreground branch. Only those genes with LRT *p*-values less than 0.05 were inferred as being positively selected.

In addition, EasyCodeML [46] was used to perform positive selection tests for the multiple-copy OGs that were significantly expanded or contracted in *N. pumilus* compared to other ladybird species by CAFE 4.2 [45] (*P* < 0.05). For most of the OGs, we selected the branch-site model with the models used for single-copy ortholog sequences above and labeled them in the *N. pumilus* clades to perform data analysis. For the lineage-specific OG in *N. pumilus* or OGs that existed in only the other four ladybirds, we used the site models with M8 vs. M7, M8vs. M8a and M2a vs. M1a as alternative and null models.

### Transcriptional regulation

*N. pumilus* preys mainly on *Icerya* and has not been reported to feed on other preys in the field. To elucidate the mechanisms underlying its feeding habits and adaptation to the natural prey *Icerya* pests. We selected fourth-instar larvae and female adults feeding on the natural prey *I. aegyptiaca* as the experimental materials and maintained under laboratory conditions (temperature: 25 ± 1 °C; relative humidity (RH): 75 ± 5%; photoperiod: 14:10 (L:D) h). Adults and larvae of *N. pumilus* were fed on *I. aegyptiaca* or not fed, respectively, for 24 h. During experiments, each individual was placed in a separate plastic Petri dish (3.5 cm diameter and 1.2 cm height). One individual was used as one biological replicate, and six adult replicates and four larvae replicates of each treatments were sequenced. The total RNA of each individual was extracted using TRIzol reagent (CWBIO, Beijing, China). RNA quality and quantity were determined using a Nanodrop 1000 spectrophotometer (Thermo Fisher Scientific, Wilmington, USA). Only RNA samples with a 260/280 ratio from 1.8 to 2.0, a 260/230 ratio from 2.0 to 2.5 and an RNA integrity number (RIN) greater than 8.0 were used for sequencing. Sequencing was performed on the Illumina HiSeq 2500 platform, generating 2 × 125 bp (Base pairs) reads. Adaptors and low-quality sequences were removed using the default settings for Trimmomatic v0.36 [68].

Initially, indexing of the reference genome and alignment of reads to the reference genome was performed using HISAT2 [73]. The aligned reads were then used for transcript quantification using StringTie v2.1.4 [75]. Genes with low expression were filtered to avoid false positives in the differential expression analysis. We used a filter method similar to that provided in the edgeR package [107]. In detail, the rows of counts of genes containing more than 4 samples with counts per million (CPM) greater than 1.0 were retained, and others were filtered. Trimmed Mean of M-values (TMM) normalization of the expression counts was conducted by the edgeR package. Then, the TMM value was used to generate the heatmaps. The coefficient of determination (*r*^2^) from Pearson’s correlation analysis was used to analyze the relationship of each sample pair based on TMM values. The regulation of gene expression in each pair of comparations was tested using the Bioconductor package DESeq2 [108], with a fold change > 2 or < 0.5 and a false FDR Q-value < 0.05 used as the criteria for defining DEGs. We used *N. pumilus* without feeding as a control to test for transcriptional regulation with feeding on the natural prey *I. aegyptiaca*. We investigated the Pfam domains that the DEGs were involved in and evaluated the statistical significance of the Pfam enrichment results by hypergeometric distribution testing using clusterProfiler package [105]. *P*-values were further adjusted for multiple testing using Benjamini-Hochberg Procedure with the cutoff of 0.05.

## Supplementary Information

**Additional file 1: Table S1.** Completeness of genome/gene set of each insect species in this study were assessed by applying the Benchmarking Universal Single-Copy Orthologs (BUSCO, Insecta set) pipeline and the expansion/contraction of gene copy numbers of orthogroups in 14 species. **Table S2.** Functional annotation of the gene set predicted from the genome of *Novius pumilus*.

**Additional file 2: Table S1.** Information on the orthologous groups identified by OrthoFinder and the *p*-value in the branch of *Novius pumilus* estimated by CAFE. **Table S2.** The genes under significant positive selection pressure (likelihood ratio test (LRT), *P* < 0.05) by branch-site model among the 711 single-copy genes of* Novius pumilus*.

**Additional file 3: Table S1.** Details of transcriptome profiling of *Novius pumilus* larvae under feeding on the nature prey *Icerya aegyptiaca* and not feeding. Expression of genes of feeding the *Icerya aegyptiaca* treatments were compared to those of the not feeding treatment in larvae. **Table S2.** Details of transcriptome profiling of *Novius pumilus* adults under feeding on the nature prey *Icerya aegyptiaca* and not feeding. Expression levels of genes of feeding on the *Icerya aegyptiaca* treatments were compared to those of the not feeding treatment in adults. **Table S3.** The RNA-Seq datas we used for genome annotation of each ladybird species. **Table S4.** The predictions and their weights used in EVM.

**Additional file 4: Figure S1.** Function of the studied genes, their accession in Pfam database and result of enrichment analysis of their upregulated DEGs of *Novius pumilus* adults in feeding on *Icerya aegyptiaca* and not feeding. Terms with Q value < 0.05 were considered as significantly enriched. **Figure S2.** Function of the studied genes, their accession in Pfam database and result of enrichment analysis of their downregulated DEGs of *Novius pumilus* adults in feeding on *Icerya aegyptiaca* and not feeding. Terms with Q value < 0.05 were considered as significantly enriched. **Figure S3.** Pipeline of genome annotation of the ladybird genomes using FunAnnotate.

## Data Availability

The raw and assembled sequenced data of *N. pumilus* were deposited in the NCBI BioProject: PRJNA626074 https://www.ncbi.nlm.nih.gov/bioproject/PRJNA626074. The RNA-Seq raw data of *N. pumilus* were deposited in the Sequence Read Archive (SRA) repository of the NCBI under accession Nos. SRR15420491 - SRR15420510 of BioProject PRJNA753304 https://www.ncbi.nlm.nih.gov/biosample/?LinkName=bioproject_biosample_all&from_uid=753304.

## Abbreviations

ABC: ATP binding cassette transporter
bp: Base pairs
BUSCO: Benchmarking Universal Single-Copy Orthologs
CAFE: Computational Analysis of gene Family Evolution
COE: Carboxylesterase
CSR: Chemosensory receptor
Gb: Gigabase pairs
GH: Glycosyl hydrolase
GILT: Gamma interferon inducible lysosomal thiol reductase
GO: Gene Ontology
GST: Glutathione S-transferase
Insect PBP: Insect pheromone-binding family, A10/OS-D
IR: Ionotropic receptor
kb: Kilobase pairs
KEGG: Kyoto Encyclopedia of Genes and Genomes
LRR: Leucin erich repeat
LRT: Likelihood ratio test
Mb: MEGABASE pairs
ML: Maximum likelihood
OBP: Odorant binding protein
OR: Odorant receptor
P450: Cytochrome P450
PFCP: papain family cysteine protease
TMM: Trimmed Mean of M-values
SNMP: Sensory neuron membrane protein
UGT: UDP-glucuronosyltransferase
