# Supplementary material for: Genomic insight into the scale specialization of the biological control agent Novius pumilus (Weise, 1892)
Source: BMC Genomics. 2022 Jan 31;23:90. doi: 10.1186/s12864-022-08299-w (PMC8805230; doi:10.1186/s12864-022-08299-w)
Supplement: Supplementary file 4 — Additional file 4: Figure S1. Function of the studied genes, their accession in Pfam database and result of enrichment analysis of their upregulated DEGs of Novius pumilus adults in feeding on Icerya aegyptiaca and not feeding. Terms with Q value < 0.05 were considered as significantly enriched. Figure S2. Function of the studied genes, their accession in Pfam database and result of enrichment analysis of their downregulated DEGs of Novius pumilus adults in feeding on Icerya aegyptiaca and not feeding. Terms with Q value < 0.05 were considered as significantly enriched. Figure S3. Pipeline of genome annotation of the ladybird genomes using FunAnnotate. [file 12864_2022_8299_MOESM4_ESM.docx]

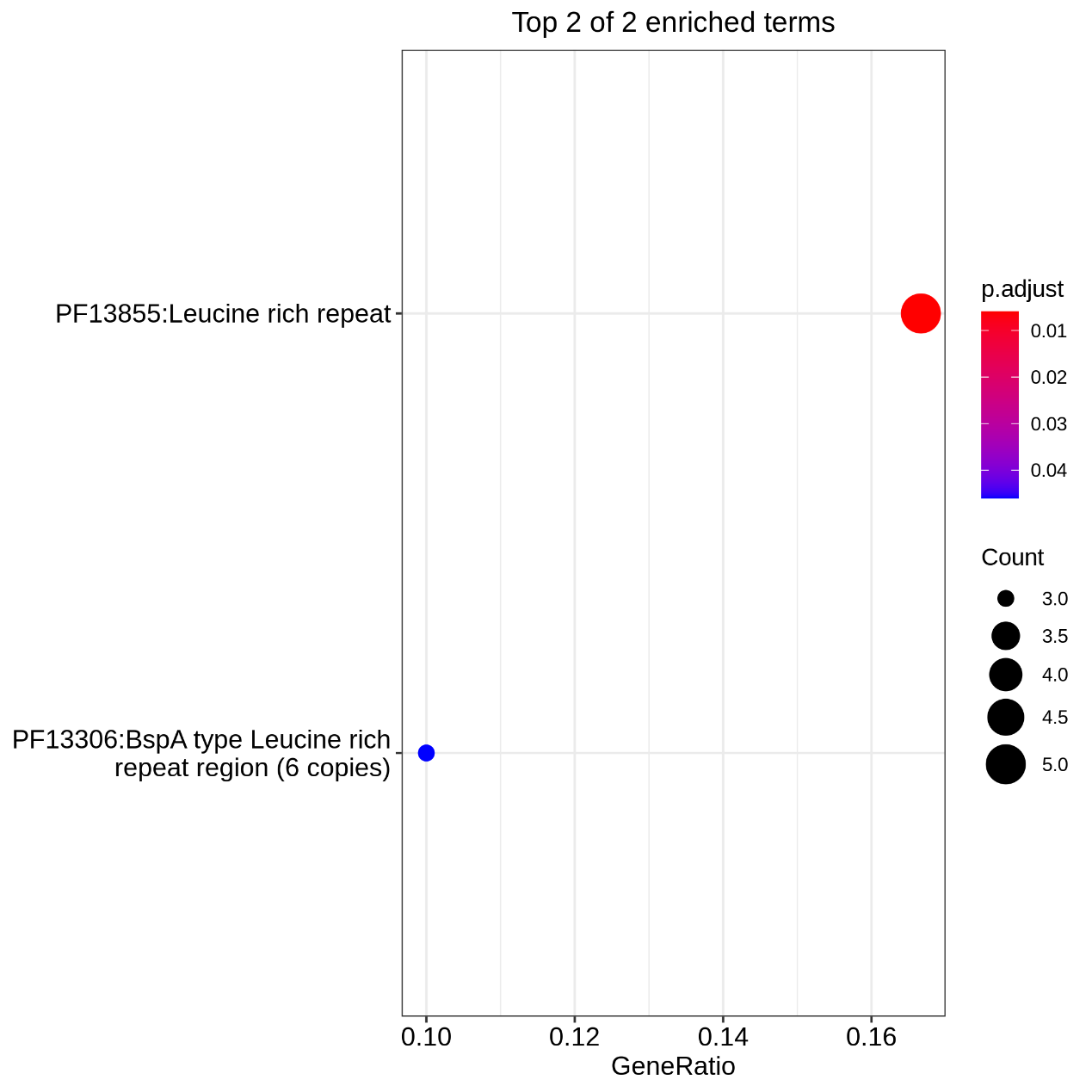


**Figure S1.** Function of the studied genes, their accession in Pfam database and result of enrichment analysis of their upregulated differentially expressed genes (DEGs) of *Novius pumilus* adults in feeding on *Icerya aegyptiaca* and not feeding. Terms with Q value < 0.05 were considered as significantly enriched.


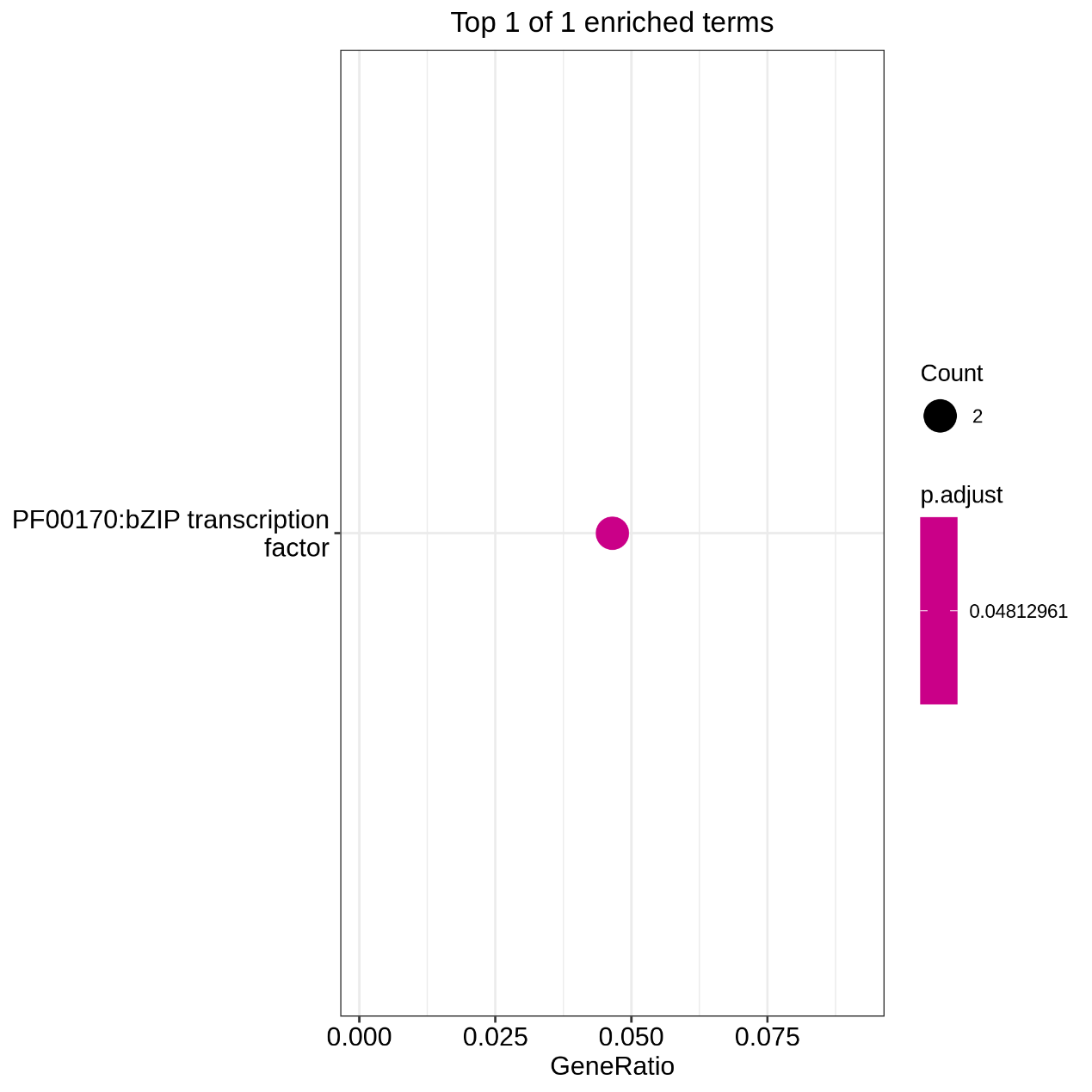


**Figure S2.** Function of the studied genes, their accession in Pfam database and result of enrichment analysis of their downregulated differentially expressed genes (DEGs) of *Novius pumilus* adults in feeding on *Icerya aegyptiaca* and not feeding. Terms with Q value < 0.05 were considered as significantly enriched.


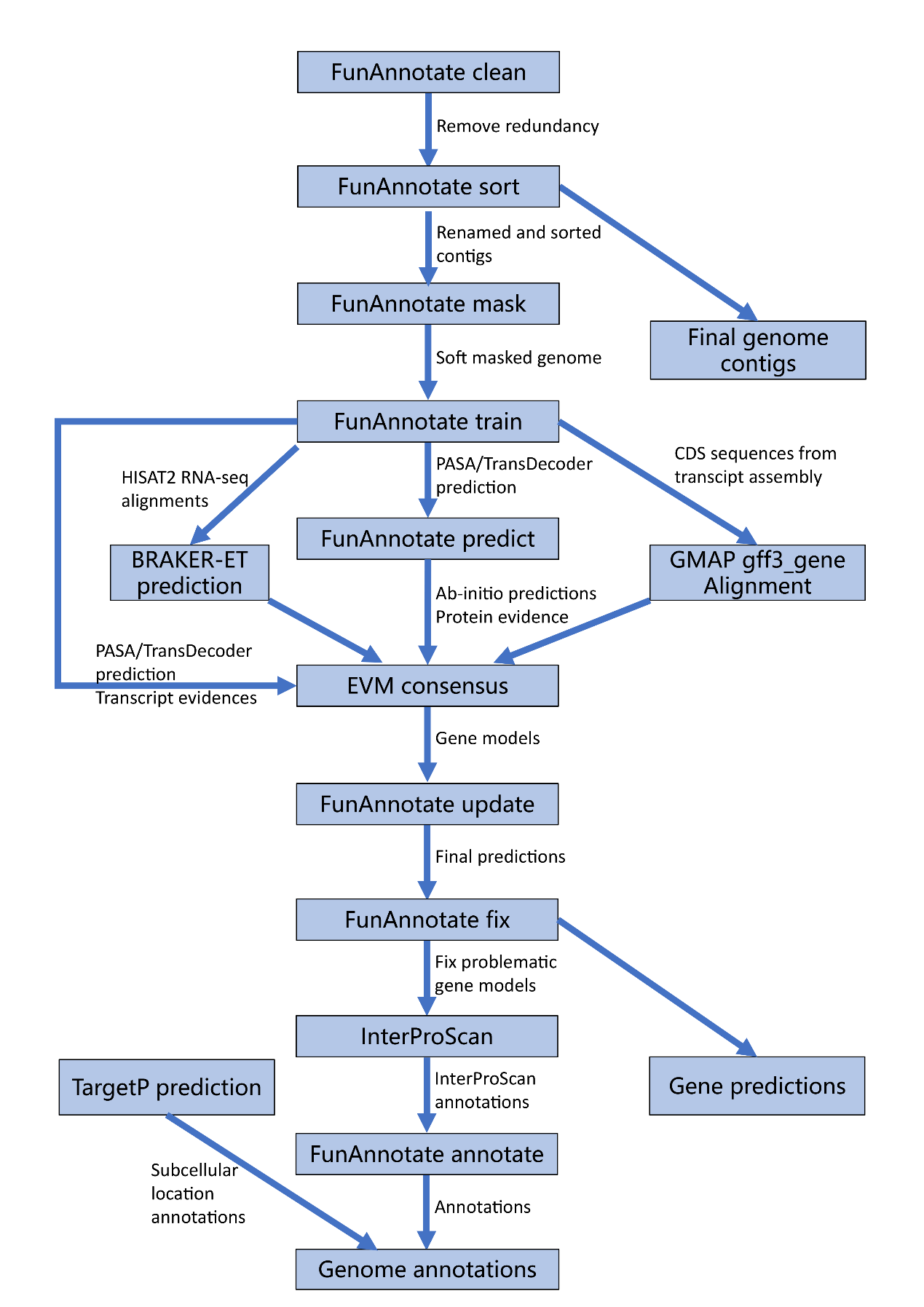


**Figure S3.** Pipeline of genome annotation of the ladybird genomes using FunAnnotate.
